# Development of Guidelines for Hospital Adoption of Wearable Telemonitoring Systems in Chronic Disease Management: Protocol for a Multiphase Mixed Methods Study

**DOI:** 10.2196/89603

**Published:** 2026-10-22

**Authors:** Janatarum Srihandono, Winny Setyonugroho, Adi Utarini

**Affiliations:** 1 Department of Health Policy and Management, Faculty of Medicine, Public Health, and Nursing, Universitas Gadjah Mada, Jl. Farmako Sekip Utara, Senolowo, Sinduadi, Mlati, Sleman, Yogyakarta, 55281, Indonesia, 62 274 545458; 2 Department of Information Technology, Universitas Muhammadiyah Yogyakarta, Bantul, Yogyakarta, Indonesia

**Keywords:** chronic disease, diabetes mellitus, guideline, hospitals, implementation science, Indonesia, technology adoption, telemonitoring, wearable devices, remote monitoring

## Abstract

**Background:**

The integration of wearable device–based telemonitoring into hospital systems presents a transformative opportunity for chronic disease management, particularly in settings such as Indonesia facing a high burden of noncommunicable diseases. However, widespread adoption is hindered by multifaceted operational barriers, including workflow integration, data management, and unclear adoption pathways, highlighting a critical need for structured, evidence-based guidance.

**Objective:**

This study protocol aims to develop a role-specific adoption guideline for integrating wearable telemonitoring systems into routine workflow in Indonesian hospitals for chronic disease management.

**Methods:**

An exploratory sequential mixed methods design is employed across 3 phases. Phase 1 involves a critical interpretive synthesis of the literature to synthesize evidence on wearable technology adoption to populate the Domain-by-Actor matrix. Phase 2 comprises qualitative semistructured interviews with 32 to 44 key stakeholders (administrators, IT staff, and clinicians) from 4 purposively selected hospitals in Yogyakarta, Indonesia, to explore contextual barriers and facilitators. Phase 3 uses a modified nominal group technique with a panel of 12 experts to build consensus and finalize the guideline.

**Results:**

As of December 13, 2025, this study is in progress. Ethical approval has been obtained. The critical interpretive synthesis (phase 1) is completed, yielding preliminary thematic data on adoption factors. Data collection for the qualitative interviews (phase 2) has commenced, with interviews conducted at 1 hospital and administrative permissions secured for 2 sites. Recruitment and data collection are ongoing.

**Conclusions:**

This protocol offers a systematic methodology to bridge the gap between digital health pilot studies and routine use in clinical operations. By mapping complex adoption factors onto an anticipated Domain-by-Actor matrix, the proposed study has the potential to guide sustainable, role-specific wearable telemonitoring integration across diverse hospital structures.

## Introduction

Technology, particularly the proliferation of consumer-grade wearable devices and the Internet of Things, has revolutionized health care delivery by enabling remote patient monitoring and decentralized care models [1,2]. The COVID-19 pandemic acted as a significant catalyst, accelerating the adoption of telemedicine and demonstrating its viability for remote care [1]. In Indonesia, this shift is now supported at the policy level, with the national health insurance agency (BPJS Kesehatan) officially using telemedicine in primary and advanced care facilities [3]. Wearable technology, with its capability for continuous, real-time data collection on digital biomarkers such as heart rate and physical activity, presents a transformative opportunity to enhance the management of chronic diseases by providing personalized feedback and promoting self-management [4,5].

This potential is critically needed in Indonesia, which faces a rapidly growing burden of noncommunicable diseases amid an aging population, placing immense strain on the national health insurance system [6,7]. Chronic conditions, such as heart disease, stroke, and diabetes, account for a substantial portion of catastrophic health care costs and profoundly impact patients’ quality of life, leading to loss of autonomy and increased dependency on family caregivers [8,9]. While Indonesia has established chronic disease management programs called PROLANIS, their implementation faced challenges including limited health care facility capacity, the requirement for physical attendance, and infrequent monitoring. Consequently, there is a clear need for more continuous and integrated care solutions [8,10].

Despite clear potential and technological advancement, the widespread and sustainable integration of wearable telemonitoring into routine hospital operations and clinical workflows remains limited [11]. Significant operational barriers hinder adoption, including concerns about the integration of patient-generated health data into existing electronic health records, the perceived increase in workload for health care professionals, unclear reimbursement mechanisms and business models, and a lack of standardized protocols for device management and training [12-14].

To analyze this multifaceted adoption landscape, this study is guided by 2 complementary frameworks, the Nonadoption, Abandonment, Scale-up, Spread, and Sustainability (NASSS) framework and the Theoretical Framework of Acceptability (TFA). The NASSS framework proposes that technology adoption is influenced by the dynamic interaction across 7 domains, namely condition, technology, value proposition, adopter system, health/care organizations, wider system, and continuous embedding and adaptation over time [15]. To gain deeper analytical depth, the TFA is nested within the organizational and adopter domains of NASSS. The TFA consists of 7 constructs offering multitemporal utility to evaluate intervention acceptability throughout its development, evaluation, and deployment phases [16]. While patient, technological, and wider system acceptability are critical in implementation research, acceptance by health care staff is a critical determinant in technology adoption. Within the NASSS framework, these professionals are situated across the adopter system and organization; for example, they may resist a technology if they feel it violates their professional code of conduct or compromises care quality [15,17]. Such phenomena are covered in one of the constructs of the TFA, labeled as “ethicality,” which is defined as the extent to which the intervention has good fit with an individual’s value system [16]. More broadly, the TFA conceptualizes acceptability as the degree to which people delivering or receiving a health care service consider it appropriate based on their anticipated or experienced cognitive and emotional responses. This focus enables the framework to guide assessments across multiple temporal perspectives, particularly prospective evaluations prior to deployment to identify potential implementation barriers early [16,18].

The TFA is selected over alternative frameworks such as the Consolidated Framework for Implementation Research (CFIR) to avoid redundancy [15,16]. The CFIR covers constructs across intervention, outer setting, inner setting, individual characteristics, and process [19]. Applying a determinant framework such as the CFIR to complement NASSS would create conceptual duplication since both models target overlapping macro-level organizational architecture. To analytically handle these framework dynamics, overlapping findings where both frameworks appear to address the same phenomenon are resolved through a hierarchical rule: data are first anchored to the specific structural NASSS domain and then coded using the most suitable behavioral TFA construct. Conversely, contradictory findings where the NASSS domain suggests one interpretation but the TFA suggests another will be treated as productive analytical friction, resolved through peer debriefing among authors, and reported transparently. The integration of the 2 complementary frameworks is shown in Figure 1.

**Figure 1.**
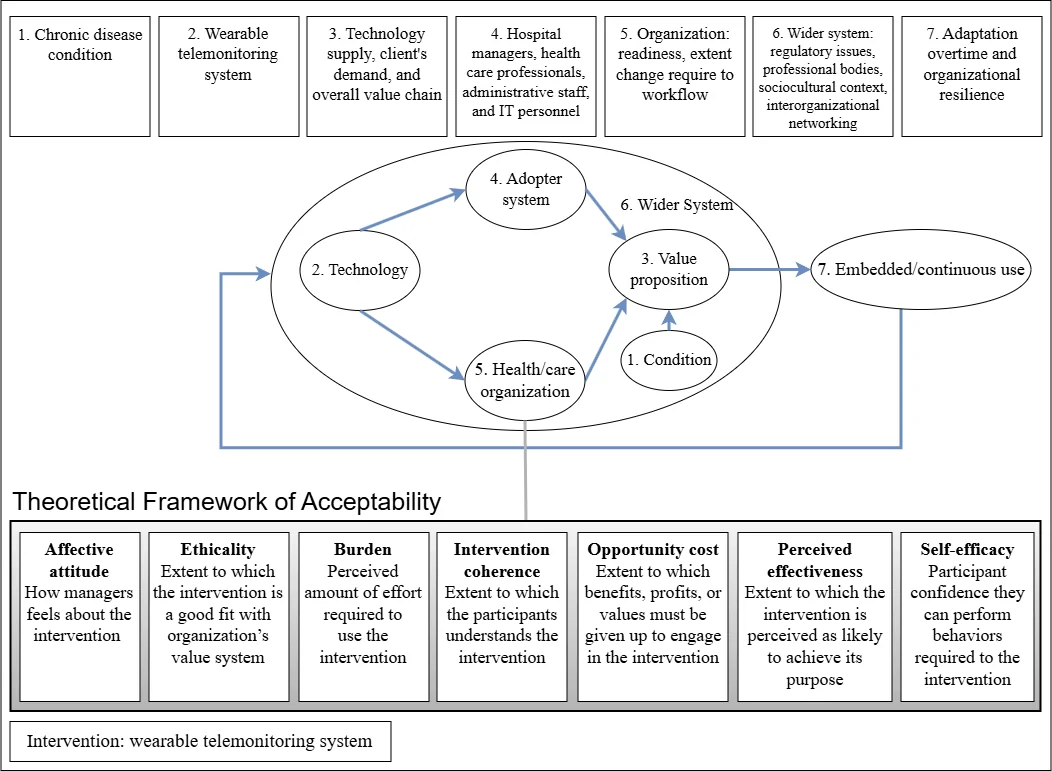
Integration of Theoretical Framework of Acceptability into the Nonadoption, Abandonment, Scale-Up, Spread, and Sustainability framework in the health/care organization domain.

This gap between a promise and practical, scaled adoption underscores a pressing need for structured guidance. Existing research often focuses on proof-of-concept, device functionality, or patient compliance, but lacks a comprehensive, actionable framework for hospital-level adoption that addresses the multifaceted barriers across technical, clinical, administrative, and financial domains [14,20,21]. Without a clear, evidence-based implementation framework, hospitals struggle to navigate these complexities, leading to failed initiatives, abandonment of technology, and an inability to systematically harness wearable technology to improve health care efficiency, reduce costs, and enhance chronic disease outcomes [15,22]. This study protocol aims to develop an evidence-based adoption guideline to facilitate the integration of wearable telemonitoring systems in Indonesian hospital settings for managing chronic disease, especially type 2 diabetes mellitus. The final guideline will be structured using the operational Domain-by-Actor matrix, serving as a sociotechnical operational framework, with the horizontal rows representing the organizational tiers and the vertical columns representing the NASSS domains. Inspired by the multilevel framework of factors influencing organizational learning by Harrison and Shortell [23], the organizational tiers are divided into 4 levels, that is, the strategic tier (responsible for director-level decisions), managerial tier (responsible for team/unit management), clinical tier (responsible for direct service delivery), and supporting tier (responsible for service delivery support). Intersecting with these rows, the vertical columns consist of selected NASSS framework domains (technology, condition, adopters, and organizations). This matrix format allows each organizational tier to identify context-specific, actionable recommendations tailored to their responsibilities within the hospital ecosystem.

## Methods

### Ethical Considerations

The study protocol has received formal ethical approval from the Medical and Health Research Ethics Committee of the Faculty of Medicine, Public Health, and Nursing, Universitas Gadjah Mada (approval number: KE/FK/1866/EC/2025). Prior to participation, all individuals involved in interviews or nominal group technique (NGT) will receive written and verbal information regarding the study’s objectives, procedures, and their rights. Additionally, site-specific approvals are being acquired for each participating institution, and no participant recruitment or stakeholder interviews will commence at any sites until all respective hospital permissions have been officially granted. Informed consent will be obtained in writing, and participants will be informed of their voluntary decision and may withdraw from the study without any consequences. Modest compensation will be provided for each participant involved. This study also ensures confidentiality and privacy. All interview transcripts and NGT notes will remain anonymous and are stored securely on password-protected platforms, including deidentification in data use during analysis and publication. To ensure participant confidentiality, access to the raw data is strictly restricted to the primary research team. It is also good to note that the wearable telemonitoring system has no intention to replace gold-standard medical devices or intervene in the management of patients’ conditions.

### Study Design

This study will employ an exploratory sequential mixed methods design, preceded by a critical interpretive synthesis (CIS) design, to develop an adoption guideline. The design comprises 3 distinct yet interconnected phases: (1) knowledge synthesis, (2) preliminary guideline development and qualitative stakeholder engagement, and (3) consensus building. This approach ensures the guideline is grounded in the existing evidence, refined by stakeholders’ perspectives, and finalized through expert consensus. Figure 2 summarizes the process and output for each phase.

**Figure 2.**
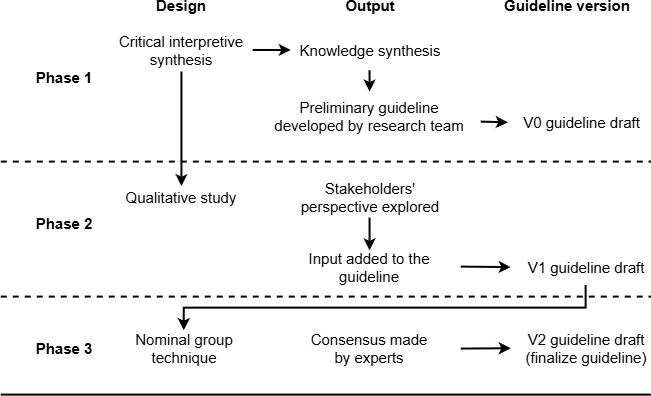
Study design, research output, and guideline development version for each study phase.

### Study Setting

The study will be conducted in Yogyakarta Special Province, Indonesia, with a life expectancy of 75.36 years in 2024, supported by 79 accredited hospitals and 121 primary care health centers. Yogyakarta has a high prevalence of type 2 diabetes mellitus, making it a relevant setting for chronic disease management innovation [24]. The research will be conducted in 4 hospitals that are selected purposively to capture diverse organizational contexts: an academic hospital owned by a public university (RSA UGM) with a 398-bed capacity; a public teaching and referral hospital with a total 867-bed capacity (RSUP dr. Sardjito) owned by the Ministry of Health; a private not-for-profit hospital (RS PKU Muhammadiyah, Gamping) with a 272-bed capacity, owned by one of the largest Muslim organizations in Indonesia; and a private for-profit hospital (RS Siloam) with a 100-bed capacity. These hospitals provide telemedicine services, indicating enthusiasm for adopting digital health innovation. The 4 hospitals with various characteristics represent different ownership, infrastructure capacity, organizational size and cultures, and business models. The study is projected to take 18 months.

### Study Phases and Procedures

#### Phase 1: Knowledge Synthesis and Preliminary Guideline Development

The first step of knowledge synthesis involves the CIS to establish a foundation for the development of the adoption guide. The CIS is selected over other knowledge synthesis methods due to the complex and multifaceted nature of the existing literature on technology adoption in health care. CIS is specifically designed to interpret and analyze diverse evidence and generate new theoretical understanding and conceptual frameworks [25]. The iterative, flexible, and dynamic approach allows for more interpretive analysis that is crucial for examining multifaceted adoption challenges [26]. The CIS process involves formulating the review question, “How do existing evidence perspectives inform the adoption of wearable telemonitoring systems for chronic disease management in hospital settings?,” which will serve as a compass to guide the inquiry. The second step consists of a comprehensive literature search across multiple databases—including PubMed, JMIR, IEEE Xplore, Cochrane, and Google Scholar—as well as conference proceedings and dissertation databases, to identify a sampling frame of potentially relevant papers [26]. This study uses the following search terms: adoption or implementation, wearable, telemonitoring or remote monitoring, chronic disease, and their derivatives as keywords. The Boolean operators “AND” and “OR” will be used to combine these terms (Multimedia Appendix 1).

Eligibility criteria are defined by content, timeframe, context, study design, and language. Papers that investigate wearable or telemonitoring systems for chronic disease management and those that address implementation or adoption challenges, facilitators, or guidelines for adopting telemonitoring technology are included (content). Concerning the timeframe, papers are eligible if published in 2015 or after, as this period captures the rise of wearable technology. The context is research undertaken in the health care setting, including hospitals. Finally, papers written in English or Indonesian using all study designs are eligible.

The third step is selecting papers purposively based on their likely relevance to offer key conceptual insights and identify the initial range of concepts related to telemonitoring adoption in chronic diseases. This initial sampling is then followed by theoretical sampling, an iterative process that operates concurrently with theory generation, where researchers dynamically refine questions and literature searches based on the emerging themes until theoretical saturation is reached [25-27]. Empirical studies concerning adoption or use of wearable telemonitoring systems in chronic diseases are selected to capture the actual implementation phenomena. We exclude articles that merely focus on the technological aspects, such as the development of devices or systems, without their implementation in patients.

The fourth step is quality appraisal adopting a critical orientation, in a broader sense of critique. Rather than applying rigid critical appraisal, where each study is judged against a predefined set of methodological standards, this approach involves deep questions about how the literature constructs problems, assumptions, and influences proposed solutions, while applying a low threshold for inclusion to maximize the contribution of papers at the conceptual level and exclude fatally flawed papers. A study is defined as fatally flawed if it fails to meet the methodological standards; specifically, it lacks clearly stated research objectives, employs an inappropriate research design for the objectives, fails to provide a clear research process, presents insufficient data to support its interpretation and conclusions, or uses inadequate and unclear methods of analysis [25]. The goal of appraisal focuses on generating key concepts relevant to the adoption of telemonitoring systems using wearable devices, thus applying flexible relevance boundaries in order to be as inclusive and comprehensive as possible.

The fifth step is data extraction. This process is a deeply interpretive and iterative process [26]. The process begins with a detailed inspection of the included papers, focusing on identifying the main arguments or themes and key conceptual contributions of each paper [25]. Investigators note the emergent themes relating to the adoption of telemonitoring technology in hospital settings, including title, objectives, publication year, country of research, study design, sample size, population, study duration, and expected outcome, which are extracted from the articles. Subsequently, findings are identified and categorized to answer the synthesis questions, which may involve findings stated or deduced based on implications presented in the literature and investigators’ insights using relevant theoretical frameworks, involving interpretive judgment beyond summarization [28]. The analysis then aims to generate a new theoretical framework or conceptual model referred to as a synthesizing argument. The key analytical technique draws heavily on techniques from primary qualitative research, particularly grounded theory, in an iterative and dynamic manner [29]. Multidisciplinary review is encouraged to introduce checks and balances and guard against a single interpretive perspective [25]. The extracted data are then sorted and categorized into themes in line with the modified NASSS and TFA frameworks. The NASSS framework serves as the primary organizing framework to map findings into the domains (ie, technology, condition, adopter, or organization), whereas TFA serves as a complementary analytical framework to further explore the adopter and/or organizational domains, as this protocol focuses on adoption in a hospital setting with 4 organizational tiers [15].

Finally, formulation of synthesis, where the concepts are constantly compared with the data to identify the relationships among them, aims to develop a synthesizing argument in the form of a coherent theoretical framework, including a network of constructs and the relationships between them. The process of formulating synthesis is logged to document reasoning or changes as investigators correlate each finding.

CIS is an interpretative methodology that was inspired by qualitative research. Dixon-Woods et al [25] recommend multidisciplinary review teams to allow a broad range of perspectives to be incorporated throughout the synthesis process in CIS. This collaborative approach establishes a vital system of “checks and balances” that prevents framing the analysis according to a single perspective. Such collaboration is beneficial during the iterative stages of refining the “compass” research question and identifying relevant literature from adjacent fields. Engaging a multidisciplinary team of authors serves as a model for verifying the reliability of the interpretive findings and is often considered a precondition for producing high-impact research.

This protocol comprises 3 authors with multidisciplinary backgrounds to represent medical doctor practitioners (JS), health technology researcher (JS), system and technology development (WS), health care service (AU), and qualitative and implementation research (AU). Throughout the process, database searching, screening, data extraction, and quality appraisal tasks will be conducted primarily by JS as part of doctoral research. Systematic searching, screening, extraction, and preliminary findings will be thoroughly discussed with WS and AU for critical review, verification, and correction. Disagreements among the team will be resolved through consensus. The process of key analytical decisions and how disagreements are resolved will be documented.

#### Phase 2: Qualitative Study for Guideline Refinement

Phase 2 aims to delve deeper and explore the complexities of telemonitoring adoption in hospital settings. This involves the process of contextualizing guidelines by translating CIS findings into actionable items. These discussions will be structured to explore adoption factors across the technological requirements, implementation procedures, and institutional considerations in correspondence to the modified NASSS framework (technology, organization, and adopters) to produce documented recommendations with actionable statements. The revealed concerns of adopters are noted for subsequent analysis through the TFA constructs. Data collection will involve semistructured individual interviews with stakeholders from 4 different hospitals with telemedicine services available. Within each hospital, informants will be selected through intensity sampling, a purposive strategy that selects information-rich cases manifesting the phenomenon of interest intensely but not extremely [30]. To capture diverse roles across hierarchical levels and professional groups, participants are recruited across 3 organizational tiers: strategic, middle, clinical, and supporting tiers. The strategic tier is represented by the hospital director or vice director of medical service (n=1), and the middle tier will be those who manage team members and understand the operational workflows, resource capabilities, and budgetary considerations, that is, head of the medical service division (n=1) and head of the IT department (n=1). The clinical tier refers to health care professionals delivering clinical services, represented by doctors and nurses (n=1‐2 persons each), while administrative staff (n=1‐2 persons) and information system support (n=1‐2 persons) will represent the supporting tier. Therefore, the tentative number of participants is 8 to 11 participants per hospital, or in total 32 to 44 participants, subject to saturation. This number may vary across the 4 hospitals as some hospitals may apply leaner organizational structures.

Data collection will continue until thematic saturation is achieved. Saturation will be assessed first by within-tier group saturation, where data will be evaluated by stakeholder tiers to specify operational perspectives related to their roles. Second, cross-group saturation will be evaluated across the entire dataset as a whole to identify full insights into adoption barriers, facilitators, and acceptability concerns. Saturation will be monitored iteratively and concurrently during data collection. As interviews progress across the sites, the research team will continuously review the new codes emerging within each tier. When 2 consecutive interviews within a tier yield no new conceptual codes, saturation for the tier is considered achieved [31]. The decision regarding saturation will be made through discussions with coauthors (AU and WS). This dual approach to considering saturation, that is, within- and cross-tier groups, aligns with the methodological model of information power proposed by Malterud et al [32], where qualitative sample size sufficiency is determined by the depth of information a sample holds rather than merely the number of informants.

The primary investigator (JS), a male doctoral student with a medical doctor practitioner background and health technology researcher, will conduct the face-to-face semistructured interviews. Having an understanding of the technical infrastructure will enable JS to conduct probing during interviews. However, to prevent the interviewer’s potential biases, neutrality will be maintained using a reflexive diary throughout data collection. The research process will be supervised by a senior female researcher with long experience as a qualitative researcher (AU). Prior to the interview, JS will introduce the study in a meeting facilitated by the participating hospitals, followed by approaching the participants individually via face-to-face meetings to create appointments for the interview. The interview will be conducted in the hospital setting for about 60 to 90 minutes and audio-recorded, with supplementary notes taken by the interviewer. The interview guide will be developed based on the CIS findings and pilot-tested for clarity before the formal data collection. The semistructured interview will explore adoption factors across the technological requirements, implementation procedures, and institutional considerations in correspondence to the modified NASSS framework domains, while any revealed concerns of adopters are analyzed through the TFA constructs.

Data analysis will follow a thematic approach, combining inductive and deductive methods. An initial deductive codebook will be developed a priori based on the modified NASSS framework domains (condition, technology, adopter, and organization) and the TFA constructs. This codebook will provide a starting point for coding while remaining open to themes emerging from the data. All interviews are transcribed verbatim, deidentified, and organized by hospital and organizational tiers. NVivo software will be used for organization, retrieval, and cross-referencing of coded data across participants. All authors will familiarize themselves with the transcripts.

The following data analysis will be carried out. First, inductive open coding will be conducted on the transcripts to identify meaning units and emergent codes grounded in participants’ perspectives. Second, the codes will then be assigned according to the predetermined categories deduced from the NASSS-TFA codebook. Those within the adopter and organization categories will be further grouped using the TFA constructs. Third, codes emerging from the inductive analysis will be reconciled with the deductive codebook. New categories may still emerge from inductive codes that do not fit within the NASSS-TFA domains. As categories should be mutually exclusive, all categories will then be reviewed until no categories overlap. Decisions made during the reconciliation process will be documented.

Themes are refined through an iterative peer debriefing process between authors. Analysis specifically maps barriers, facilitators, and opportunities for wearable telemonitoring system adoption to the modified NASSS framework (technology, adopter system, health/care organization, and condition) to contextualize findings. Within the organization domain, TFA serves as a deductive coding and interpretative lens. The inductively derived themes are then compared against TFA constructs as an organizing template to categorize underlying adoption barriers, facilitators, and opportunities. Consistent with the tier-actor–based approach, the analysis will prioritize cross-stakeholder comparison to identify “who does what” in the adoption of telemonitoring systems. This cross-stakeholder analysis will inform the tiered structure of the guideline to address the specific responsibilities, concerns, and acceptability mechanisms relevant to each organizational tier. The final synthesized themes, illustrated with representative quotes, will inform the guideline’s development by comparing the existing matrix results from phase 1 and identifying gaps; new role-specific recommendations will be formulated, or local context conflicts with global findings will then be enhanced using the same standardized syntax established in phase 1.

#### Phase 3: Consensus Building for Guideline Finalization

The final phase of this study employs a modified NGT, a structured consensus-building process and controlled small-group process ideal for synthesizing expert judgments to develop clinical practice guidelines [33-37]. The process is reported following the ACCORD (Accurate Consensus Reporting Document) reporting guideline for consensus methods [38]. NGT is selected over alternative consensus methods because its structured format prevents single individuals from dominating the discussion. Various perspectives of experts allow a multidisciplinary panel of experts to transparently assess and reconcile differing assumptions within the forum [37,39]. Moreover, NGT allows for listing issues during the development and deployment of eHealth applications. It is considered an adequate technique for conceptual validation, serving to verify the potential suitability, sustainability, and practicality for real-life decision-making [40,41].

A purposively selected panel of approximately 12 experts will be invited to participate in the NGT through consensus among all authors. To capture a comprehensive range of perspectives, the NGT panel will comprise hospital directors, managers of medical services, finance, and information technology, as well as health service experts from academia. A person is considered an expert if they possess a high level of knowledge or lived experience relevant to the topic, in line with methodological guidance for the expert panel [42]. Hospital directors are eligible if they are currently working or former hospital directors with 5 years of experience at the strategic level and are familiar with telemedicine services. Managers at the managerial level are eligible if they are currently working or formerly worked in hospital management, have 3 years of experience in a decision-making role within a hospital organization in medical services, administration/finance, or IT, and are familiar with telemedicine. A health service expert is a teacher/researcher in health care/hospital management and registered as a lecturer at a recognized university in Indonesia. Panel recruitment will be started with purposive sampling, followed by snowball sampling, where initially selected participants recommend eligible colleagues. Panel participants will be reimbursed for their time.

The NGT process will be implemented using the Indonesian language, led by a steering committee. The steering team consists of the first author (JS), supervised by AU and WS, with research experience in health care (AU) and technology (WS). Prior to commencement, a briefing document will be prepared by JS and disseminated to all expert panel members by email 1 week in advance, as not all participants are familiar with the topic.

The NGT will follow 5 key stages [33]: (1) introduction and explanation, where V1 guideline as a result of knowledge synthesis and qualitative study is a matter to discuss and the core question for improvement is clarified, that is, “We have developed the guideline based on the evidence synthesized (CIS) and stakeholders’ feedback (qualitative study). The materials are not piloted for the NGT prior to the NGT process. What is needed to improve the adoption of wearable telemonitoring guidelines for hospitals to manage chronic conditions?” This step also gives an opportunity to state the aim, which is development of an adoption guideline based on prior phases’ findings, and to clarify the question; (2) silent generation of ideas, where participants individually reflect on and document their suggestions to enhance the guideline; (3) round-robin sharing, where each participant states 1 idea at a time for transcription without discussion; this stage ensures every participant has an equal opportunity; (4) clarification, where the group discusses each idea for understanding and to group similar concepts; and (5) voting by participants. The steering committee will not be included in the decision of consensus.

The ranking stage will involve 2 rounds of private voting using a 9-point Likert scale (1=highly inappropriate, 9=highly appropriate) to evaluate both appropriateness and feasibility of each recommendation. Following established consensus methodologies, a predefined threshold will be used to determine agreement and govern the final Domain-by-Actor matrix. Items achieving a median score of 7 to 9 paired with majority agreement of more than 50% plus 1 expert will be considered “appropriate” and included; items with a median of 1 to 3 will be considered “inappropriate” and discarded; and items with a median of 4 to 6, or those with a median of 7 to 9 that fail to reach more than 50% majority agreement threshold, will be flagged for further discussion and modification in the second voting round, allowing for reevaluation and refinement of the consensus qualitatively [43,44]. If the modified items clear the majority agreement threshold on the second and final vote, they will be included; if they fail to achieve more than 50%, they will be systematically excluded to protect the integrity of the framework. After each round of voting, the steering committee will present the result of the voting round. The research phases of the study design are summarized in Figure 3.

**Figure 3.**
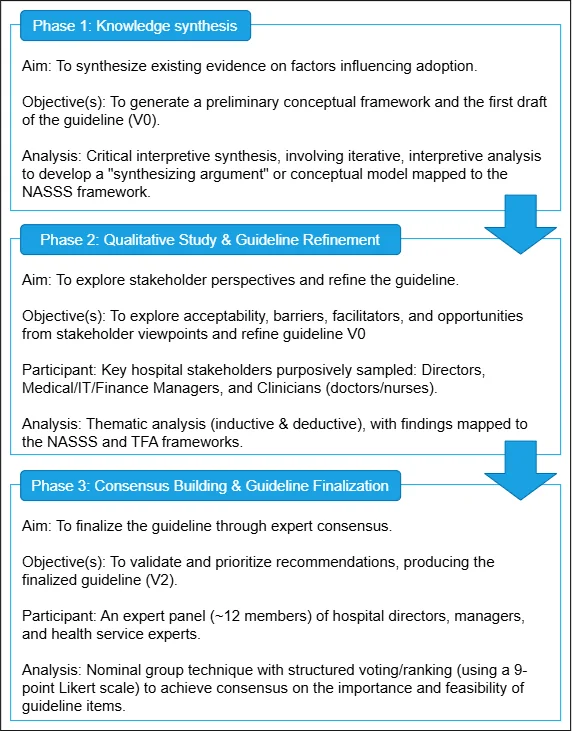
Phases of developing guidelines for adopting wearable telemonitoring systems. NASSS: Nonadoption, Abandonment, Scale-Up, Spread, and Sustainability; TFA: Theoretical Framework of Acceptability.

The multiphase research design is chosen to address the development of adoption guidelines, as it builds a foundation based on existing evidence, is contextually grounded by a qualitative study, and is expert-validated. The design follows an exploratory sequential mixed methods approach to develop adoption guidelines. The synthesized foundation through CIS is contextualized in the qualitative study to refine the guideline. All 3 phases are essential, but the qualitative phase holds the highest importance, as it is meant to contextualize global findings into an Indonesian context relevant to the target setting, which will also be the content of expert validation in the NGT. The integration occurs at 3 points. First, after the CIS, the results are used to develop the semistructured interview guide. This ensures data collection by JS, supervised by AU, is grounded in the existing evidence. Second, after the qualitative study, themes are integrated to produce version 1 of the guideline, combining the CIS result guideline into a more contextualized version of the draft (V1); this point is completed by JS and all 3 authors. The third integration point is when the draft guideline after the qualitative study is presented to the NGT panel and is reviewed by an expert panel, refined, and prioritized through consensus. The definitive endpoint of this study is reached when the matrix is populated with role-specific, actionable recommendations synthesized and validated through the 3 study phases.

## Results

This study protocol has been approved by the Medical and Health Research Ethics Committee of the Faculty of Medicine, Public Health, and Nursing, Universitas Gadjah Mada. The results presented herein pertain to the current progress of the study setup. As of the submission of this protocol on December 13, 2025, the study is actively progressing. The initial knowledge synthesis (phase 1), designed to inform the phase 2 interview guide, has been completed. This synthesis has yielded preliminary thematic data on adoption factors. Building on this, data collection for the qualitative study (phase 2) has commenced. To date, semistructured interviews have been conducted with a telemedicine service manager and an administrative staff member at 1 participating hospital, and transcription of these interviews is underway. Administrative permissions have been secured for 2 targeted hospitals, and we are currently coordinating with a second hospital to finalize its specific institutional ethical clearance requirements. Further participant recruitment for phase 2 is ongoing.

The study is designed to generate several key outputs that collectively address the research gap in wearable telemonitoring adoption. The primary output will be a finalized, expert-validated implementation guideline (version 2), providing hospitals with a practical, step-by-step framework for integrating wearable devices into chronic disease management. This guideline will be directly informed by 3 core deliverables: a systematic summary of existing evidence on adoption factors derived from the CIS; a rich qualitative analysis of stakeholder perspectives, barriers, and facilitators; and a prioritized list of consensus-based recommendations generated through the NGT. These outputs are intended for dissemination through peer-reviewed publications and direct engagement with health care stakeholders to facilitate real-world application.

## Discussion

This study provides an essential procedure for addressing the systemic organizational challenges of integrating wearable telemonitoring into routine hospital workflows for managing chronic disease. The anticipated primary finding of this study is an operational Domain-by-Actor matrix that maps hurdles to specific roles of individuals within the hospital organization. The matrix is populated with actionable operational recommendations on who-does-what in adopting wearable telemonitoring, aligned with the organizational tiers and selected domains of the NASSS framework. This ensures clarity on each role and incorporates domains in unison to adopt wearable telemonitoring systems in hospitals, moving beyond technological or clinical focus alone. The operational matrix guide is expected to address a critical gap in the digital health landscape between pilot studies and scaled implementation by providing a roadmap [14,45].

While other studies have investigated the use of telemonitoring systems, few studies have focused specifically on guides for developing guidelines for wearable or telemonitoring use. Szeto et al [14] developed implementation strategies for wearable activity trackers used to enhance physical activity and patient outcomes. Yet their work primarily focuses on wearable technology, neglecting the potential for integrating wearable devices into telemonitoring systems. Similarly, Smuck et al [20] identified factors for successful implementation of wearable technology in health care settings but overlooked the potential for integrating wearable devices into remote monitoring systems. Meanwhile, Gokalp et al [46] demonstrated a proof of concept for the potential of telemonitoring systems in health care delivery to collect vital signs and lifestyle data to support independent living for frail older adults with chronic diseases. In contrast, this study explicitly focuses on the use of wearables as a telemonitoring medium specifically designed for chronic disease management within routine hospital operations.

Furthermore, guidelines should be based on available evidence, which requires evaluating, summarizing, and making recommendations based on the available evidence, involving stakeholder engagement as recommended by the World Health Organization and the National Institute for Health and Care Excellence [47]. By adapting these rigorous institutional principles to the evolving landscape of digital health, this study utilizes a CIS to synthesize knowledge, a qualitative study to involve stakeholders, and the NGT to transform fragmented empirical evidence into a validated, role-specific Domain-by-Actor matrix.

The chosen multiphase design of sequentially integrating knowledge synthesis, qualitative exploration, and expert consensus, the methodology ensures that the resulting guideline is not only evidence-based but also contextually grounded and feasible for addressing the complexities of adopting emerging technologies in hospital settings. Using the modified NASSS framework ensures that the complexity of the adoption process—spanning technology, organization, the wider system, and adopters—is systematically addressed, moving beyond a simplistic focus on the technology itself [15,48]. This approach is aligned with best practices in implementation science, which emphasize the need to develop strategies based on a deep understanding of local barriers and facilitators [49]. Furthermore, by grounding this empirical exploration across 4 highly distinct hospital archetypes (academic public, tertiary referral, private nonprofit, and private for-profit), the protocol ensures that diverse organizational cultures are captured. Ultimately, the primary strength of this design lies in its anticipated operational output, translating these multidimensional insights into a role-specific Domain-by-Actor matrix that clearly defines “who-does-what” operational workflows across complex institutional hierarchies.

Several potential limitations are acknowledged. First, the study is conducted within the specific context of the Indonesian health care system, which may limit the immediate generalizability of the guideline to other countries with different funding models and regulatory environments. However, the methodological approach and the core NASSS-based domains are likely to be transferable. Second, the rapidly evolving nature of wearable technology poses a challenge and a risk of the guideline becoming outdated. This is mitigated by focusing the recommendations on fundamental adoption principles, processes, and organizational changes rather than on specific, fast-changing device specifications. Finally, the success of the NGT is dependent on the expertise and engagement of the selected panel, a risk that will be mitigated through purposive sampling to ensure diverse and relevant expertise.

While numerous studies demonstrate the clinical efficacy of telemonitoring, few provide health care administrators and IT managers with a structured, actionable roadmap for adoption. By offering practical recommendations on key operational hurdles, such as workflow integration, staff training, IT infrastructure, and business model development, this study, if successful, has the potential to reduce failed implementation attempts, optimize resource allocation, and accelerate scalable adoption of remote patient monitoring in Indonesia and similar settings. The study will contribute to the broader field of health services research by providing a replicable methodology for translating digital health evidence into practical tools for health care systems.

Future research directions will involve empirically testing the deployment of this Domain-by-Actor matrix in clinical rollouts to evaluate its long-term impact on institutional deployment. Future researchers can adapt this protocol to develop adoption guides for other rapidly emerging health technologies, such as AI and decentralized clinical trial systems, across diverse global health infrastructures.

For optimum practical utility of the Domain-by-Actor matrix, the dissemination strategy will be targeted according to organizational tiers, across health facilities and their stakeholders, including the participating hospitals in this study. In addition to scientific publications, the final Domain-by-Actor matrix will be tailored into engaging information designed to attract the strategic tiers in collaboration with local hospital associations. The introduction will be designed to build awareness and simplify adoption of emerging technologies to assist and enhance health care delivery. This “hook” will serve as a gateway for interested health care facilities or technology developers to initiate deeper operational matrix discussions.

In conclusion, this protocol establishes a methodological path to bridge the operational gap separating wearable telemonitoring pilot studies from sustained, scaled adoption within routine hospital workflows. Through sequentially integrating a CIS, diverse empirical fieldwork across 4 distinct hospital types, and structured validation via the NGT, this research moves beyond a purely technological or clinical focus. The anticipated operational Domain-by-Actor matrix addresses a major gap in implementation science by translating complex, multidimensional barriers into practical, role-specific considerations across all organizational tiers.

## Supplementary material

10.2196/89603 Multimedia Appendix 1 Critical interpretive synthesis process example.

## Abbreviations

ACCORD: Accurate Consensus Reporting Document
CFIR: Consolidated Framework for Implementation Research
CIS: critical interpretive synthesis
NASSS: Nonadoption, Abandonment, Scale-Up, Spread, and Sustainability
NGT: nominal group technique
TFA: Theoretical Framework of Acceptability
