# Supplementary material for: Development of Guidelines for Hospital Adoption of Wearable Telemonitoring Systems in Chronic Disease Management: Protocol for a Multiphase Mixed Methods Study
Source: JMIR Res Protoc. 2026 Oct 22;15:e89603. doi: 10.2196/89603 (PMC13690334; doi:10.2196/89603)
Supplement: Supplementary file 1 [file resprot-v15-e89603-s001.docx]

Appendix 1: Method details

Database search: PubMed, JMIR, IEEE Xplor, Cochrane, and Google Scholar

Search strategy:

((((adopt*) OR (implement*)) AND ((wearable*) OR ("activity tracker*"))) AND ((((telemonitoring) OR (remote monitoring)) OR ("remote monitoring")) OR ("distance monitoring"))) AND ((((("chronic disease") OR (chronic disease)) OR ("chronic illness")) OR ("chronic condition")) OR (chronic condition))

Example

| Date | Database | Strings | Filters | Result |
| --- | --- | --- | --- | --- |
| 9/2/2025 | Pubmed | ((((adopt*) OR (implement*)) AND ((wearable*) OR ("activity tracker*"))) AND ((((telemonitoring) OR (remote monitoring)) OR ("remote monitoring")) OR ("distance monitoring"))) AND ((((("chronic disease") OR (chronic disease)) OR ("chronic illness")) OR ("chronic condition")) OR (chronic condition)) | from 2015/1/1 - 2025/8/31 | 49 |

Example PRISMA-style flow diagram:
